# Real-Time MRI Monitoring of GelMA-Based Hydrogel-Loaded Kartogenin for *In Situ* Cartilage Regeneration

**DOI:** 10.3389/fbioe.2022.940735

**Published:** 2022-07-22

**Authors:** Hanyuan Zhang, Weijun Fang, Tingting Zhao, Huabing Zhang, Liang Gao, Jingya Li, Rujing Wang, Weiping Xu

**Affiliations:** ^1^ Institute of Intelligent Machines, Chinese Academy of Sciences, Hefei, China; ^2^ Department of Biological Physics, University of Science and Technology of China, Hefei, China; ^3^ Department of Orthopedics, The First Affiliated Hospital of Anhui Medical University, Hefei, China; ^4^ School of Basic Medical Sciences, Anhui Medical University, Hefei, China; ^5^ Hua Tuo Institute of Medical Innovation (HTIMI), Wuhan, China; ^6^ Sino Euro Orthopaedics Network, Berlin, Germany; ^7^ The First Affiliated Hospital of USTC, Division of Life Sciences and Medicine, University of Science and Technology of China, Hefei, China

**Keywords:** kartogenin, cartilage regeneration, dopamine, magnetic resonance imaging (MRI), hydrogels

## Abstract

The cartilage has poor ability to mount a sufficient healing response. Herein, kartogenin (KGN), an emerging stable non-protein compound with the ability to recruit bone marrow mesenchyme stem cells (BMSCs) to promote chondrogenic differentiation, was grafted onto dopamine-Fe(III) chelating nanoparticles, followed by involving a gelatin- and dextran-based injectable hydrogel to mimic the extracellular matrix to promote cartilage repair. The *in vitro* results demonstrated that KGN underwent long-term sustained release behavior and availably promoted the deep migration of BMSC cells in yielding hydrogels. Furthermore, *in vivo* New Zealand white rabbits’ cartilage defect model repairing results showed that cartilage defect obtained significant regeneration post operation in the 12th week, and the defect edge almost disappeared compared to adjacent normal cartilage tissue. Meanwhile, the T_2_-weighted magnetic resonance imaging (MRI) property resulting from dissociative Fe (III) can significantly monitor the degradation degree of the implanted hydrogels in the defect site. This integrated diagnosis and treatment system gives insight into cartilage regeneration.

## Introduction

Cartilage has a rather limited ability for self-repair and regeneration due to its unique avascular microstructure and low cell density (Zhang et al., 2020a; Zhang et al., 2020b). Although the common treatment methods for cartilage injury in clinical, including marrow stimulation, autografts and matrix-induced autologous chondrocyte implant, can alleviate the pain, they also have limited problems such as ectopic cartilage damage, insufficient donors and immune rejection (Yan et al., 2020a; Satapathy et al., 2020; Wang et al., 2020; Yin et al., 2020; Naghizadeh et al., 2021). Hence, it is still a challenge to obtain natural hyaline cartilage and restore its function. Current studies utilized three-dimensional scaffolds to fill defects to suppress cartilage deterioration development caused by the peak force values on the uneven surface (Zhou et al., 2017; Liu et al., 2020a; Rogan et al., 2020; Yao et al., 2020). In addition, the scaffolds provide a mimetic extracellular matrix (ECM) for chondrocytes (Huang et al., 2015; Yang et al., 2019a). However, due to the inherent low density of chondrocytes, it is difficult to achieve effective cartilage regeneration only by providing chondrocytes with a mimicking ECM (Zhang et al., 2019; Benmassaoud et al., 2020; Dai et al., 2020). Although some researchers make attempts to promote repair by cell injection or the integrated cell-scaffold resulting from 3D printing, these approaches also encounter limitations, including the appropriate number of cells, immune rejection, and the risk of infection caused by bacteria and viruses (van Buul et al., 2011; Abdollahiyan et al., 2020). Therefore, it is particularly important to choose an effective signal stimulation to evoke the self-repair of cartilage.

In recent years, the emerging small molecule kartogenin (KGN) has high stability *in vitro*. It can recruit BMSCs to home to cartilage sites and further induce BMSCs to differentiate into chondrocytes, which has inspired researchers’ interest to use it in cartilage diseases (Zhang et al., 2017; Yang et al., 2019b; Yan et al., 2020b). However, the related study demonstrated that single KGN injections in the joint hardly promoted cartilage regeneration owing to the loss of KGN due to the effect of blood circulation (Xu et al., 2015). Although coating KGN on the surface of nanomaterials can achieve an improved sustainable release effect, the simple physical interactions can still easily cause obvious burst release, which can barely accomplish the long-term repair process (Yin et al., 2017; Sun et al., 2018).

Another important problem is that owing to the long-term repair process, it is necessary to observe the degree of repair intermittently in clinic and adopt some external intervention for the regulation of a treatment scheme to obtain the desired effect. Therefore, it is a key and urgent challenge to integrate the function of monitoring materials in a real-time and non-invasive manner. Magnetic resonance imaging (MRI), which has deep penetration capabilities, has served as a safe imaging tool for monitoring molecular changes with high resolution, the absorption states of implanted materials, and the remodeling of regenerated tissue (Kotecha et al., 2013; Ukai et al., 2015). It is also widely and effectively applied in clinical investigations (Chen et al., 2013). In the past, MRI has been utilized to visualize and assess tissue-engineered constructs due to its superior distinguishing capabilities for soft tissue (van Buul et al., 2011).

Hydrogels based on natural biomaterials have been widely used in the study of hard tissue repair, such as bone and cartilage (Zhou et al., 2018). They can mimic the ECM and serve as a carrier for *in situ* drug release. Gelatin and dextran are two of the most used biomaterials for constructing the hydrogel for biomedical use. They both have excellent biocompatibility, abundant chemical groups for modification, and biodegradability. Researchers used the two chemical-modified biomaterials to construct double crosslinked hydrogel and realized the *in situ* application (Zhou et al., 2018; Zhao et al., 2022; Zhou et al., 2022). Moreover, hydrogels can stabilize and uniformly disperse nanoparticles, which can be chosen as an ideal carrier for drugs (Liu et al., 2020b).

**SCHEME 1:**
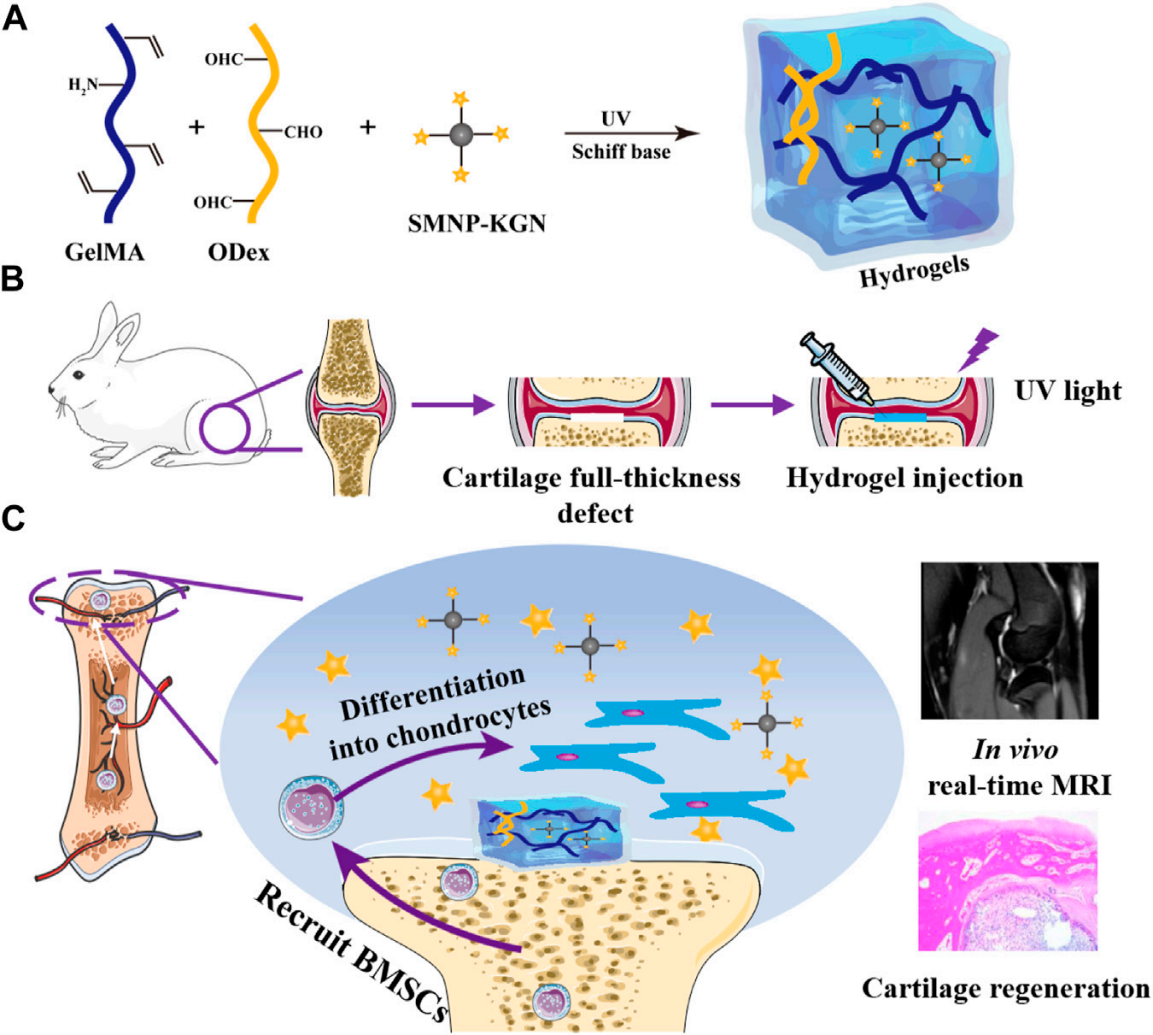
Schematic diagram of the research. **(A)** Hydrogel; **(B)** full-thickness defect surgical procedure; **(C)** GelMA/ODex/SMNP-KGN hydrogel recruits BMSC and promotes its differentiation into chondrocytes.

Based on the above background, KGN is a stable chemical component compared to protein, cells or growth factors, which allows KGN to work long term *in vivo*. One issue that needs be addressed is the development of suitable carriers to achieve sustained release of KGN. Fe (III) has long been used in MRI monitoring, and nanoparticles combined with dopamine can enhance its biocompatibility. In this research, the dopamine-Fe (III) chelating nanoparticles covalently bound to KGN and dropped into hydrogel was designed to realize the *in situ* long-term release. The prepared hydrogels can effectively fill the irregular cartilage defects, the mechanical strength of which can be adjusted by changing the construction ratio. Released KGN can effectively recruit BMSCs to the internal hydrogel and induce them to differentiate into chondrocytes. Fe (III) can effectively provide significant T_2_-weighted imaging, which can monitor the hydrogel state and regenerated cartilage in a real-time and non-invasive manner (Scheme 1).

## Materials and Methods

### Materials

Gelatin (Gel) from porcine skin was purchased from Sigma-Aldrich (Shanghai, China). Dextran (Dex, Mw = 70 KDa) was purchased from J&K (Beijing, China). Methacrylic anhydride, sodium periodate, ethylene glycol, dopamine hydrochloride, ferric chloride hexahydrate (FeCl_3_ 6H_2_O), kartogenin (KGN) and photo-initiator 2-hydroxy-4’-(2-hydroxyethoxy)-2-methylpropiophenone (Irgacure 2959) were purchased from Aladdin Industrial Corporation (Shanghai, China). BMSCs were purchased from Guangzhou Chuangseed Biomedical Materials Co., Ltd. All other chemical reagents were purchased from Sigma-Aldrich and used without further purification.

### Synthesis and Characterization of GelMA and ODex

GelMA was synthesized according to the previously described procedure (Camci-Unal et al., 2013). Briefly, 10 g of gelatin was dissolved in 100 ml of PBS at 50^o^C with continuous stirring. Then, 8 ml of methacrylic anhydride was added to the gelatin solution and stirred for 3 h at 50^o^C. The reaction solution was diluted with 300 ml of PBS and then dialyzed against distilled water (molecule weight cut-off = 12–14 kDa) for 1 week at 40^o^C to remove unreacted reagents. The final product was obtained by lyophilizing at −80^o^C.

Oxidized dextran (ODex) was synthesized as follows: 1 g of dextran was dissolved in 10 ml of purified water to obtain 10% (w/v) solution, and then oxidized with 2 ml of sodium periodate with a concentration of 100 mg/ml. The reaction was carried out in the dark for 20 h at room temperature and the theoretical oxidation was about 20% (Zhu et al., 2018). Ethylene glycol was added to the reaction solution to stop the oxidation process. The resulting solution was then dialyzed exhaustively against deionized water (molecule weight cut-off = 7–12 kDa) and lyophilized at −80^o^C to obtain the final product.

The successful synthesis of final product GelMA and ODex was characterized by Fourier Transform Infrared Spectrometer (FT-IR, Bruker, VERTEX 70, Germany) with a resolution of 4 cm^−1^. The potassium bromide (KBr) tablet method was used for preparing test samples and a blank KBr tablet was used as background.

### Synthesis and Characterization of MRI Contrast Agent SMNP and Therapeutic Agent SMNP-KGN Nanoparticles

As for the synthesis of contrast agent synthetic melanin nanoparticles (SMNP) (Li et al., 2016), 45 mg of dopamine hydrochloride and different amounts of ferric chloride hexahydrate was fully dissolved in 130 ml of deionized water, stirring at room temperature for 1 h. Subsequently, 20 ml of tris buffer was quickly injected into the above solution. It was observed that the color of solution immediately turned red and gradually turned black after 0.5 h. After stirring for another 1.5 h, the targeted SMNP nanoparticles were separated by centrifugation and washed three times with deionized water.

The SMNP-KGN nanoparticles were prepared by amidation: 20 mg of hydrophobic KGN powder was dissolved in 20 ml of dimethyl sulfoxide (DMSO) solution, and then 200 mg of SMNP nanoparticles were added to the KGN solution and sonicated for 20 min to obtain a homogeneous mixture. Next, the mixture was shaken (120 r/min) at 37^o^C in the shaker for 24 h. The precipitated SMNP-KGN nanoparticles were collected by centrifuging (8000 r/min) and lyophilized at −80^o^C. The successful synthesis of SMNP-KGN was characterized by (FT-IR, Bruker, VERTEX 70, Germany). The morphology and diameter of the resulting SMNP-KGN were characterized by TEM (H-800; Hitachi, Japan) and DLS (Zetasizer Nano ZS, Malvern, the United Kingdom).

### The Preparation and Characterization of Gel-Dex/SMNP-KGN Hydrogels

The hydrogel pre-solution was obtained by dissolving GelMA and ODex in PBS buffer at different compositions with 0.1% (w/v) photo-initiator I2959 and placed at 37°C. The GelMA solution was fixed at a final concentration of 5% (w/v) and the concentration of ODex was varied. The mass ratio of GelMA and ODex ranged from 1/9 to 9/1. Next the prepared SMNP-KGN nanoparticles were added and sonicated to obtain homogeneous mixing. Hydrogel was finally obtained by UV (365 nm) crosslinking.

The rheological experiments of the prepared hydrogels were carried out in strain-controlled mode on a rotary rheometer (Kinexus Pro^+^, Malvern, United Kingdom) with a parallel plate (20 mm) at room temperature. The strain and frequency were selected from the linear viscoelastic region, which were 1% and 1 Hz separately. The dynamic frequency sweep experiments were carried out in the range of 0.1–10 Hz at 1% strain to identify the linear viscoelastic range of the hydrogel. The GelMA/ODex hydrogels’ morphology were observed using a scanning electron microscope (SEM, LEO1530 VP, 5 kV electron beam; Philips, Netherlands) after sputter coating with gold.

### *In Vitro* Release Behavior of KGN

*In vitro* release behavior of KGN was evaluated as follows: the hydrogel containing SMNP-KGN was immersed in 3 ml of PBS buffer (pH = 7.4) at 37^o^C. At specified time intervals, 1 ml of incubation solution was taken, and residual solution was supplemented with 1 ml of PBS. The amount of KGN released was determined by UV spectrophotometry and the absorption peak of KGN was 278.4 nm.

### *In Vitro* MR Imaging Stability

The MR images were acquired on a Bruker 7.0 T magnet with Avance II hardware equipped with a 72 mm quadrature transmit/receive coil. T_1_ contrast was determined by selecting regions of interest using the software ParaVision version 5.1. The parameters for 7.0 T MRI are TR = 750.0 ms, TE = 12.6 ms, echo = 1/1, FOV = 6.91/3.12 cm, slice thickness = 2 mm, nex = 2 mm, matrix = 256 × 116.

### *In Vitro* Proliferation and Migration Performance of BMSCs

BMSCs proliferation was examined with Cell Counting Kit-8 (CCK-8) at 450 nm on days 1, 3, and 5 after seeding. The hydrogel samples (1.0 g) with different compositions of Fe (III) were incubated in 10 ml of DMEM at 37°C with shaken (100 rpm) for 24 h, the hydrogels were taken out and the DMEM culture medium was obtained as hydrogel extracts. BMSCs were seeded on 48-well plates with 5,000 cells per well. After BMSCs adhered to the wall of 48-well plate, 200 μL of different hydrogel extracts were added to each well, and then the 48-well plate was cultured at 37°C in an incubator containing 5% CO_2_ atmosphere. At the selected time point, the culture medium was replaced by CCK-8 working solution and cultured at 37 ^o^C for another 1h, the OD value at 450 nm was read by microplate reader (SpectraMax® M5, Molecular Devices, United States) following the instructions in the manual. To evaluate the cell migration performance of composite hydrogels, BMSCs were seeded on the surface of the hydrogel samples in a 6 well plate at the concentration of 2.5×10^5^ per well for 3 days, and then washed by PBS to remove unadhered cells. The different top surface was observed and taken by microscope after cutting. Migrated cell dots were quantitatively evaluated using Image-Pro Plus (v.6.0) software.

### *In Vitro* Characterization of the Ability of BMSC Differentiate Into Chondrocytes

The expression of glycosaminoglycan (GAG), type I collagen and type II collagen in BMSCs co-cultured with the hydrogel extracts were measured to characterize the potential of BMSCs to differentiate into chondrocytes.

A 1,9-dimethylmethylene blue (DMMB) dye binding assay was utilized to detect the GAG content. The samples were collected at days 7, 14, and 28 and digested with papain solution (1 mg ml^−1^ papain in 0.1 M phosphate buffer with 5 mM l-cysteine hydrochloride and 5 mM EDTA) for 18 h at 60^o^C. After centrifugation at 1000 *g* for 15 min, 20 μL of supernatant from each specimen was mixed with 200 μL of DMMB dye (16 mg of DMMB in 1 L of water containing 3.04 g of glycine, 2.37 g of NaCl, and 95 ml of 0.1 M hydrochloric acid) for 30 min at 37^o^C. The GAG content was standardized using shark chondroitin 6-sulfates.

The DNA content was measured with Hoechst 33258 diluent and normalized to a certified calf thymus DNA standard. Absorbance and fluorescence values were immediately determined after the incubation time using a SpectraMax^®^ M5 multimode microplate reader (Molecular Devices). The cartilage-specific gene (collagen I and collagen II) expression level of the GelMA/ODex, GelMA/ODex/SMNP and GelMA/ODex/SMNP-KGN hydrogel co-cultured with BMSCs for 14 days were evaluated by real-time PCR.

### Rabbit Cartilage Defect Model Construction

All animal procedures were approved by the Laboratory Animal Center of Anhui Medical University. Twenty-seven adult male New Zealand white rabbits (2.5–3 kg, 5–6 months old) were fed for 1 week to adapt to the environment before surgery. The rabbit was anesthetized by injecting 3% (w/v) pentobarbital (1 ml/kg) through the ear vein and put in the supine position. The hair on the joint was removed and the surgical site then disinfected with iodophor. The skin and surface tissue along the lateral side of the patella was cut to expose the knee joint. A drill with a core diameter of 4 mm was used to create a full-thickness cartilage defect with a diameter of 4 mm and a depth of 4 mm in the center of the joint. Subsequently, the hydrogel pre-solution was added on the defect and crosslinked using UV light. After the hydrogel formed, the skin was sutured layer by layer and disinfected. Penicillin was injected in the first 3 days after the surgery to prevent infection. After 6 and 12 weeks of treatment, the articular cartilage was collected and fixed with 4% paraformaldehyde.

For histological staining, the fixed articular cartilage was decalcified with 10% ethylenediaminetetraacetic acid EDTA for 3 months until softened, then dehydrated and embedded in paraffin. The samples were cut into 5 μm sections for further staining. H&E, Masson, toluidine blue, and PAS staining were carried out to assess the regeneration of cartilage.

### *In Vivo* MRI Evaluation

All animals were anesthetized for MRI observation after surgery at 0, 6 and 12 weeks. The parameters for 7.0 T MRI are TR = 750.0 ms, TE = 12.6 ms, echo = 1/1, FOV = 6.91/3.12 cm, slice thickness = 2 mm, nex = 2 mm, matrix = 256 × 116.

### Statistical Analysis

The data are expressed as the mean standard deviation of at least 3 parallel samples. All statistical analysis were performed using SPSS software (ver. 16.0; SPSS Inc., United States). Significance analysis is obtained through one-way ANOVA, and at *p* < 0.05 were considered statistically significant: (*) for *p* < 0.05, (**) for *p* < 0.01, (***) for *p* < 0.001.

## Results and Discussion

### The Synthesis of Hydrogel Precursor GelMA and ODex and MRI Imaging-Therapeutic Agents SMNP-KGN Nanoparticles

The matrix occupies 95% of the total volume of cartilage, and the organic components in a cartilage matrix are mainly proteins, such as collagen. As one of the collagen derivatives, gelatin exhibits unique properties such as similar biological properties to collagen, lower antigenicity and desired biocompatibility. However, due to the excessively fast degradation rate and insufficient mechanical strength, its application in hard tissue repair is limited (Levett et al., 2014).

**FIGURE 1:**
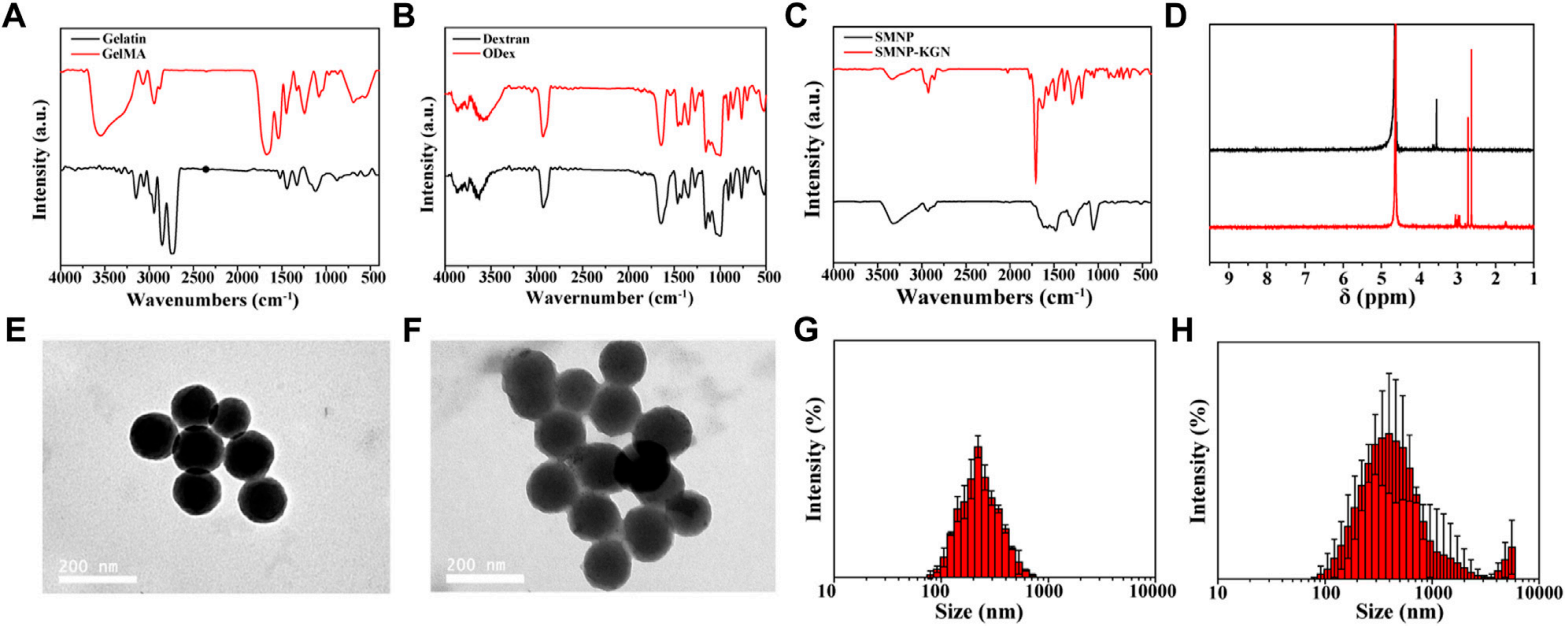
Chemical construction of synthesized materials and characterization of nanoparticles. **(A)** FT-IR spectrum of gelatin and GelMA; **(B)** FT-IR spectrum of dextran and ODex; **(C)** FT-IR spectrum of SMNP and SMNP-KGN; **(D)** ^1^H NMR spectrum of SMNP and SMNP-KGN; TEM image of **(E)** SMNP and **(F)** SMNP-KGN nanoparticles; hydration diameter statistics of **(G)** SMNP and **(H)** SMNP-KGN obtained by dynamic light scattering.

In this study, gelatin was modified by methacryloyl and served as a candidate of functioned hydrogels. The successful synthesis of GelMA was demonstrated by the FT-IR absorption peaks at 1634 cm^−1^ (amide I band), 1530 cm^−1^ (amide II band) and 1446 cm^−1^ (amide III band), corresponding to the stretching vibration of C=O bond, bending vibration of -NH bond and plane vibration of -CN and -NH bonds, respectively (Figure 1A). Although GelMA alone can form hydrogel, this hydrogel is completely formed by a chemical cross-linked network and generally has poor fatigue resistance. Articular cartilage, as a connection between bones, needs to withstand cyclic pressure, so only using GelMA as hydrogel cannot meet actual needs. Double cross-linked network hydrogels can effectively solve the problem of poor mechanical properties of single network hydrogels.

Dextran has the similar structure to natural chondroitin and has recently been applied in cartilage repair (Jin et al., 2010; Wang et al., 2017). It can be modified by a variety of chemical methods, among which the most common modification method is sodium periodate oxidation to obtain aldehyde groups. Aldehydated dextran can easily form hydrogels with amino-rich organics through a Schiff base. This dynamically reversible Schiff base crosslinked hydrogel also plays an important role in the field of biomedicine.

Previous research indicates that oxidized dextran (ODex) can form Schiff bases with unreacted amino groups in GelMA, and the GelMA/ODex hydrogel has better fatigue resistance than GelMA after crosslinked by UV light (Liu et al., 2020b). This kind of interpenetrating polymer network (IPN) can dissipate external energy well, so that the hydrogel still maintains good mechanical properties after being subjected to multiple compression cycles. Therefore, ODex is introduced into the interaction with GelMA to produce a hydrogel with double crosslinks, thereby improving the mechanical properties of hydrogel that matches natural cartilage. The successful synthesis of ODex was proved according to the FT-IR spectrum and the characteristic adsorption peak of ODex at 1722 cm^−1^ was attributed to the vibration of the aldehyde group (Figure 1B).

SMNP, a new type of nanoparticle based on dopamine, maintains the good biocompatibility of dopamine and at the same time has a high Fe (III) ion content, which has great potential as an MRI contrast agent. Through the amino groups on the surface of SMNP, KGN can be easily grafted to the surface of SMNP by amide reaction, thereby prolonging the retention time and exerting a long-term effect in the body. Figure 1C showed the successful synthesis of SMNP-KGN. The emerging peak at 1710 cm^−1^ and weakened peak at 1535 cm^−1^ was ascribed to the vibrations of carboxyl groups of KGN and amino groups of SMNP, respectively. The ^1^H NMR spectrum also shows the successful synthesis of SMNP-KGN (Figure 1D). The morphology and diameter of SMNP and SMNP-KGN was characterized through TEM and DLS. SMNP appeared as a sphere with an average diameter of 130 mm (Figures 1E,G). It was found the shape of SMNP-KGN was not significantly changed but the diameter of the complex increased slightly compared to SMNP (Figures 1F,H).

### Rheological Properties and Morphology of GelMA/ODex Hydrogel

**FIGURE 2:**
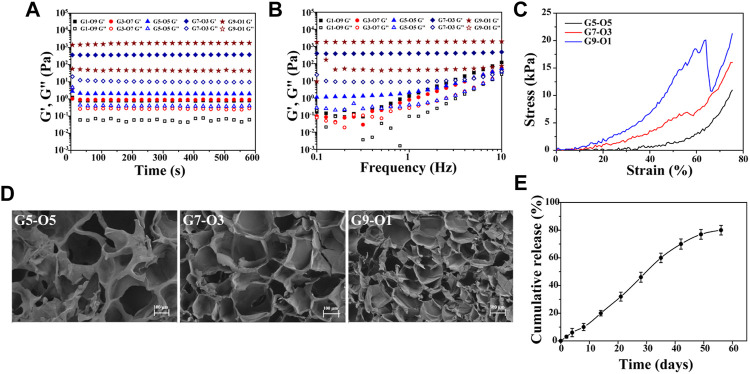
**(A)** Time-sweep sequence and **(B)** frequency sweep of hydrogel with different mass ratio; **(C)** strain-stress curve of G5-O5, G7-O3, and G9-O1 hydrogel; **(D)** SEM images of cross-sections of three hydrogels. **(E)** Release curve of KGN obtained from GelMA/ODex/SMNP-KGN hydrogel.

Hydrogel was regarded as the solid material with apparent viscoelasticity reflecting viscosity and elasticity by compressional deformation that frequently occurs in the cartilage repair. Rheological behavior of various components of hydrogels was conducted (Figures 2A,B). The results showed that the storage modulus (G’) and the viscous modulus (G”) of all hydrogels remained stable, indicating the hydrogel was fully formed. But the storage modulus increased over the proportion of GelMA increasing. For instance, the storage modulus at the maximal value was 1401 Pa for G9-O1, 366.8 Pa for G7-O3, 2.7 Pa for G5-O5, 1.035 Pa for G3-O7, and 1.218 Pa for G1-O9, indicating that the addition of GelMA was pivotal to improve the storage modulus (Figure 2A). It is worth noting that G3-O7 and G1-O9 did not form hydrogel, because the higher concentration of ODex affected the photo-crosslinking of GelMA, so these two groups were not used in the following tests. In addition, there was no significant change in storage modulus after applying a shearing force ranging from 1 to 10 Hz, but the viscosity modulus showed the changing trend over the various content of GelMA and ODex, especially the G5-O5, G3-O7 and G1-O9 group (Figure 2B). Previous literature has reported that hydrogels with storage modulus about 1,000 Pa are more conducive to protecting and maintaining the chondrocyte phenotype (Liu et al., 2018), thus the hydrogel that was prepared was suitable for cartilage regeneration.

A static compression test was conducted to further analyze the mechanical properties of three different hydrogels. As shown in Figure 2C, the stress was increased as GelMA concentration increasing. However, the G9-O1 hydrogel with the highest content of GelMA failed to dissipate the huge external pressure in time due to the higher cross-linking density of C=C double bonds and the low content of Schiff base bonds and ruptured under 60% strain. This phenomenon was also observed in the study of Liu et al. Pure GelMA hydrogel has higher compression stress due to the tighter covalently cross-linking between C=C double bonds (Liu et al., 2020b).

The morphology of hydrogels with various ratios of GelMA was observed by SEM (Figure 2D). The results showed all hydrogels exhibited similar interconnected pore morphology. The interconnectivity of the macro-pores of the hydrogels for cartilage regeneration is essential to assure cell seeding or cell invasion from subchondral bone (Mok et al., 2018). Subsequently, the pore size and quantity decreased, and the smooth pore wall became thick over the GelMA increasing. The average pore size of the G7-O3 hydrogel was about 100 μm, which could support chondrocyte growth. In addition, it can be inferred that pore size was significantly correlated with the degree of crosslinking between molecular chains. The crosslinking density became higher with the increase of G7 content. Therefore G7-O3 was applied in pivotal proportion for further research according to the above results.

### The Potential of Hydrogels as MRI Contrast and Cartilage Therapeutics Agent

Further, the *in vitro* release behavior of KGN loaded in 0.4% Fe (III) labeled hydrogels was investigated (Figure 2E). It was observed that the release was slow in the first 10 days, and then accelerated in the medium term, and the release trend flattened out eventually. The release behavior can be inferred that the crosslinking point between the macromolecular chain in the hydrogels was gradually hydrolyzed and broken, and then the macromolecules were decomposed into small molecules. The deconstruction of hydrogel accelerated the release of SMNP-KGN nanoparticles; thus, a higher release speed could be seen from day 10 to day 40. The cumulative release of KGN reached 80% on the 56th day, which was close to the release rate obtained by directly coating the KGN in PLGA microspheres according to previous literature that reported an effective cartilage repair effect (Shi et al., 2016).

**FIGURE 3:**
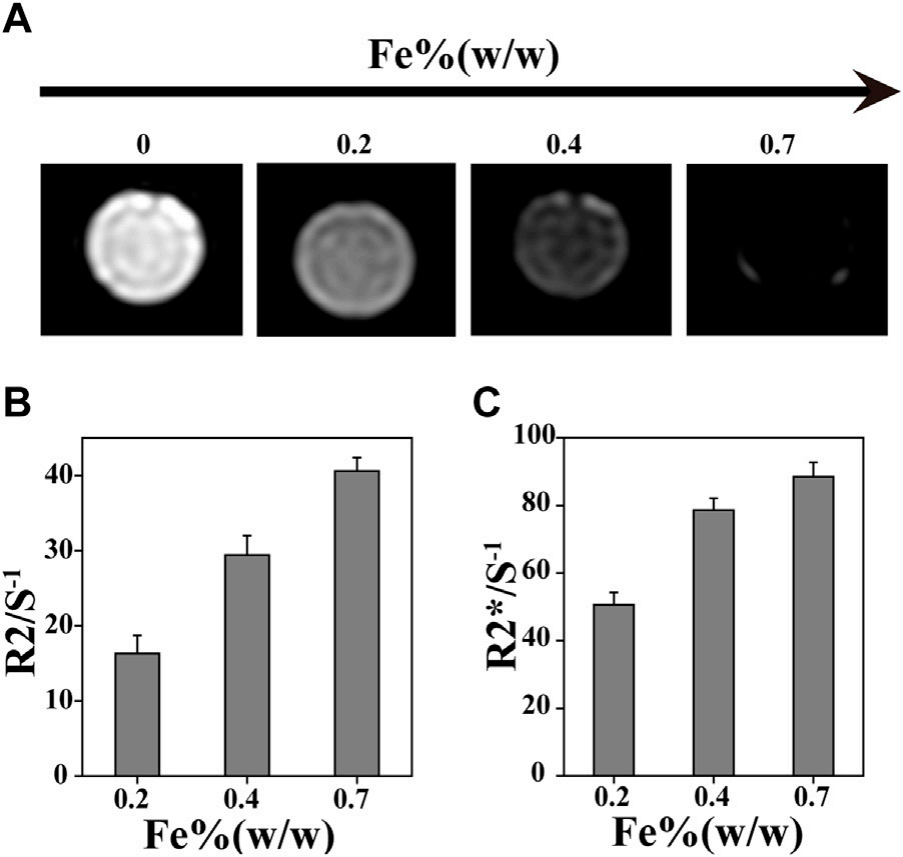
*In vitro* MR imaging ability. **(A)** MRI with different Fe concentration. **(B)** R2/S^-1^ and **(C)** R2*/S^-1^ value obtained from MR images.

*In vivo* real-time non-destructive monitoring of tissue repair is very important for guiding clinical medication. MRI can effectively observe cartilage structure, and the SMNP that was prepared is a new type of contrast agent with great potential for MRI. Hydrogels were labeled by SMNP-KGN with various content of Fe (III), and then conducted for T_2_-weighted MR images. It was found that 1/T_2_ value showed an obvious increase with the increase of Fe (III) content resulting in hyperintense image in the labeled group, and in reverse hypointense in the blank group, indicating that Fe (III)-chelated nanoparticles can enhance T_2_-weighted MR images (Figures 3A–C).

**FIGURE 4:**
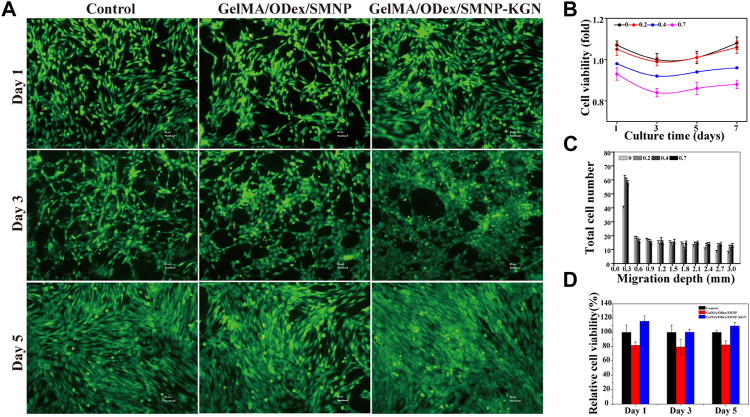
Cytocompatibility of hydrogels. **(A)** Live/dead staining of BMSCs cultured with hydrogel extracts; **(B)** cell viability of BMSCs cultured with SMNP with different Fe content; **(C)** migration depth of SMNP-KGN with different Fe (III) content; **(D)** cell viability of GelMA/ODex/SMNP and GelMA/ODex/SMNP-KGN hydrogel.

### *In Vitro* Proliferation and Migration of BMSCs Cultured With Hydrogel

To verify the biocompatibility of hydrogel, CCK-8 was used to detect the cell viability of BMSCs co-cultured with the hydrogel extract, and the cells were stained with a live-dead staining kit. Figure 4Bfig4 shows that the higher content of Fe (III) increased the cytotoxicity of SNMP nanoparticles, but 0.2% Fe contained in SMNP did not show obvious cytotoxicity. KGN has an important effect to recruit BMSCs to differentiation into chondrocyte. Cell starch assay was conducted to assess the promoting migration ability of SMNP-KGN. A significant migration depth of 0.3 mm of BMSCs appeared in the presence of SMNP-KGN with different Fe content; low migration appeared in the control group in return (Figure 4C). But when the migration depth exceeded 0.3 mm, there was no significant difference in the migration rate of BMSCs. The phenomenon could be ascribed to the effect of KGN that had the ability of protecting chondrocyte and promoting the migration of BMSCs as previously reported. Meanwhile, it is proved that the Fe content used in this study does not inhibit cell proliferation and migration. SMNP containing 0.2% Fe also behavior the MRI contrast ability and it was therefore chosen for the following study. Previous studies demonstrated that Fe concentrations higher than 25 μg Fe/mL showed an inhibiting effect on chondrogenesis of hBMSCs (Chen et al., 2018); the concentration of Fe (III) in SMNP-KGN labeled hydrogel was lower than the reported value, thus the hydrogel that was prepared would not affect the chondrogenesis of hBMSCs.

The cytotoxicity of GelMA/ODex hydrogels containing SMNP and SNMP-KGN was further studied. In the live/dead staining image (Figure 4A), BMSCs showed a good spread morphology, and no obvious dead cells (red) were seen, and the living cells (green) were in good condition. The CCK-8 assay results demonstrated though GelMA/ODex/SMNP hydrogel had a certain inhibitory effect on cell proliferation, but the addition of KGN can significantly protect cells and accelerate cell proliferation (Figure 4D).

### *In Vitro* Ability of Hydrogel to Induce BMSC to Differentiate Into Chondrocytes

**FIGURE 5:**
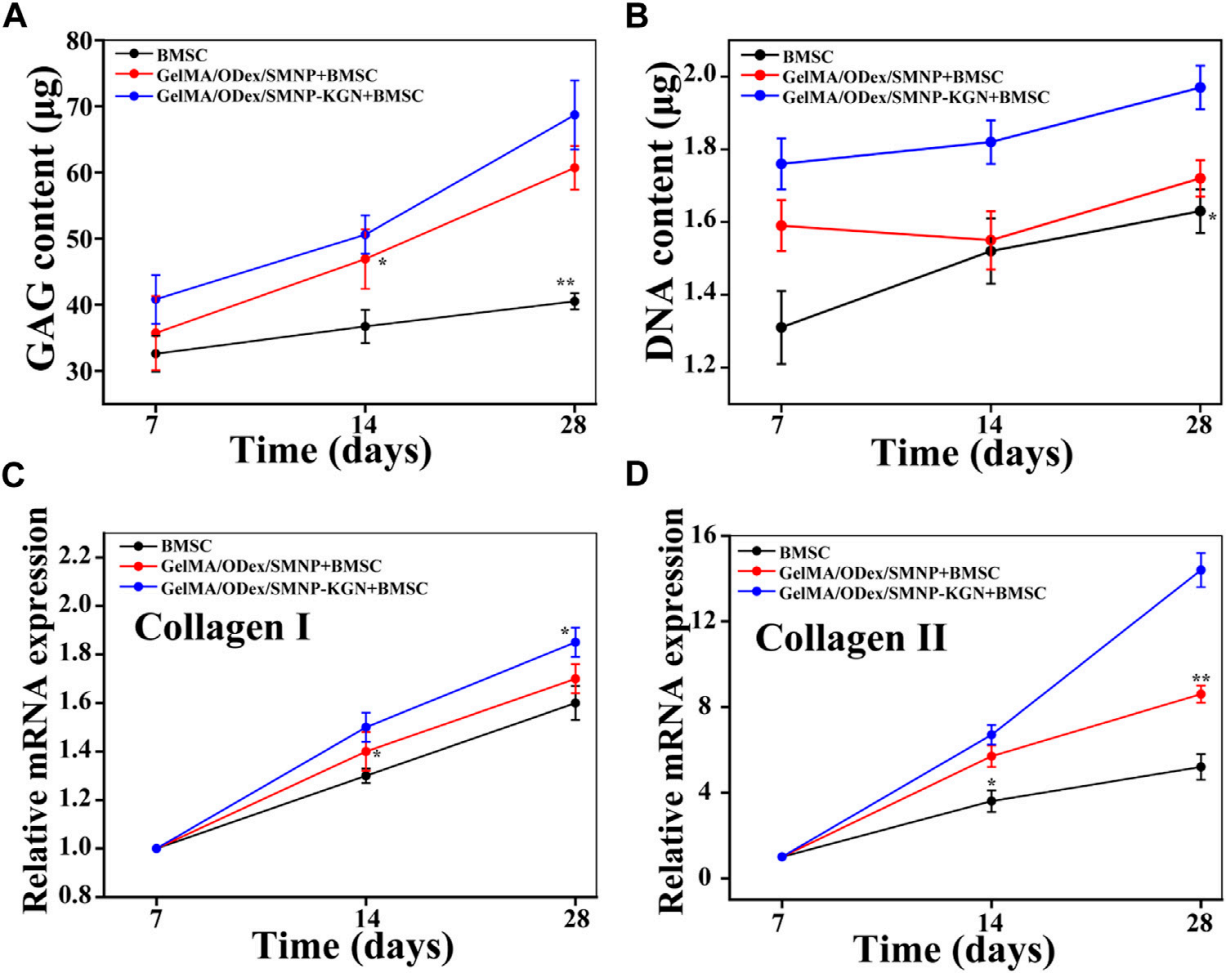
**(A)** GAG and **(B)** DNA content of cartilage matrix deposition after BMSCs co-cultured with hydrogel. Relative mRNA expression of **(C)** collagen II and **(D)** collagen I.

Next,:BMSCs were co-cultured with hydrogels to analyze the ability of hydrogel that induce BMSCs to differentiate into chondrocytes. The deposited cartilage matrix and the relative expression of cartilage matrix coding genes was investigated. Compared with the GelMA/Odex hydrogel, the GAG secretion content in the SMNP containing the hydrogel cultured group was up regulated and the expression level in KGN-SMNP containing the hydrogel cultured group was the highest. The secretion rate in the latter 14 days in the SMNP containing hydrogel was higher than that in the first 14 days, while the control group (GelMA/Odex hydrogel) only showed a slightly increasing trend (Figure 5A). The total glycosaminoglycan (GAG) contents were normalized to the DNA content and showed a robust and sustained increase (Figure 5B).

Collagen is one of the main components of cartilage, among which hyaline cartilage mainly contains collagen II (fibrocartilage mainly contains collagen I). It was determined the content of collagen I and collagen II expressed by BMSCs after co-cultured with hydrogel. As shown in Figures 5C,D, compared with the control group, the expression of collagen II in the GelMA-Odex/SMNP-KGN was significantly upregulated over time. The expression of collagen I in each group remained basically unchanged and was significantly lower than that of collagen II, indicating that the hydrogel has the potential to promote the differentiation of BMSCs to form hyaline cartilage *in vitro*, rather than forming fibrocartilage.

### *In Vivo* Full-Thickness Defect Cartilage Regeneration Assessment of Hydrogel

To evaluate the effect of hydrogel in promoting cartilage regeneration, we caused full-thickness damage to the articular cartilage of rabbits and injected the hydrogel *in situ* to completely fill the damaged area to evaluate the promotion effect of cartilage regeneration.

**FIGURE 6:**
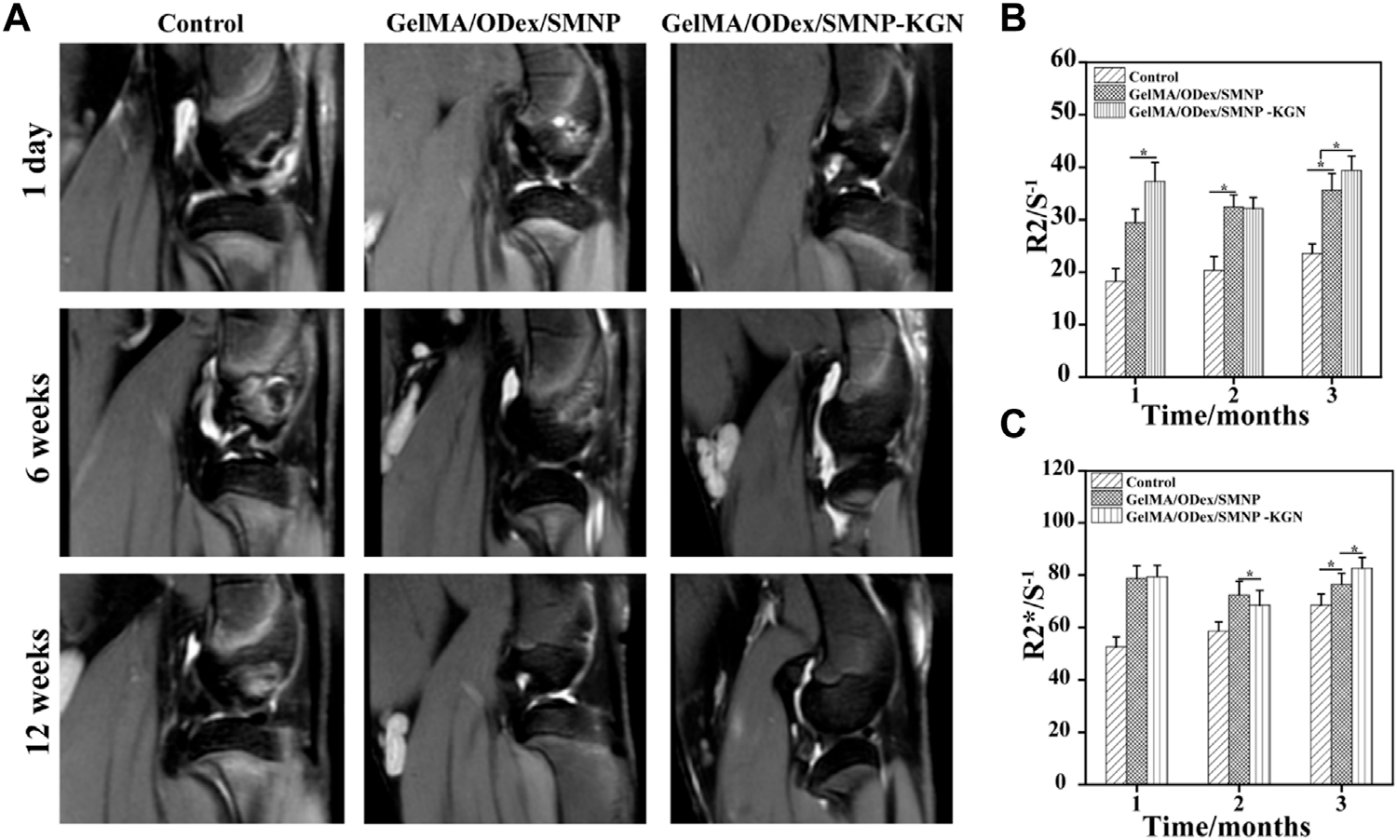
**(A)** *In vivo* T2-weight MR image of articular cartilage. **(B)** R2/S-1 and **(C)** R2*/S-1 value obtained from MR images.

SMNP labeled hydrogels were implanted in cartilage defect and monitored by MRI (Figure 6A). As shown, hyperintense image appeared in all the groups incipiently and SMNP labeled hydrogels showed more remarkable light signal compared to the control group, which was mainly ascribed to water and Fe (III) enhancing the T_2_-weighted MR images. At the 6th week, the control group kept the hyperintense, whereas the experiment groups showed weak hyperintense and strong hypointense. Combining the results of gross observation, it can be inferred that severe inflammation has not relieved over time in the control group, however the other groups treated with hydrogels emerged a repaired effect, along with hydrogels degradation that caused the loss of contrast agent, thus decreasing 1/T_2_ value. In addition, it can be found that more obvious hypointense and higher R2 and R2* value was detected in the hydrogels group with KGN, indicating that the accelerated deposition of calcium ions happened at the defect site enhancing the T_2_-weight imaging (Yang et al., 2019b). At 12 weeks, GelMA-ODex/SMNP-KGN group showed a strong shadow similar to the surrounding normal cartilage tissues, which demonstrated that KGN speeded up the process of cartilage regeneration, and that the complex hydrogels mostly degraded after the *in vivo* implantation for 12 weeks (Figures 6B,C).

**FIGURE 7:**
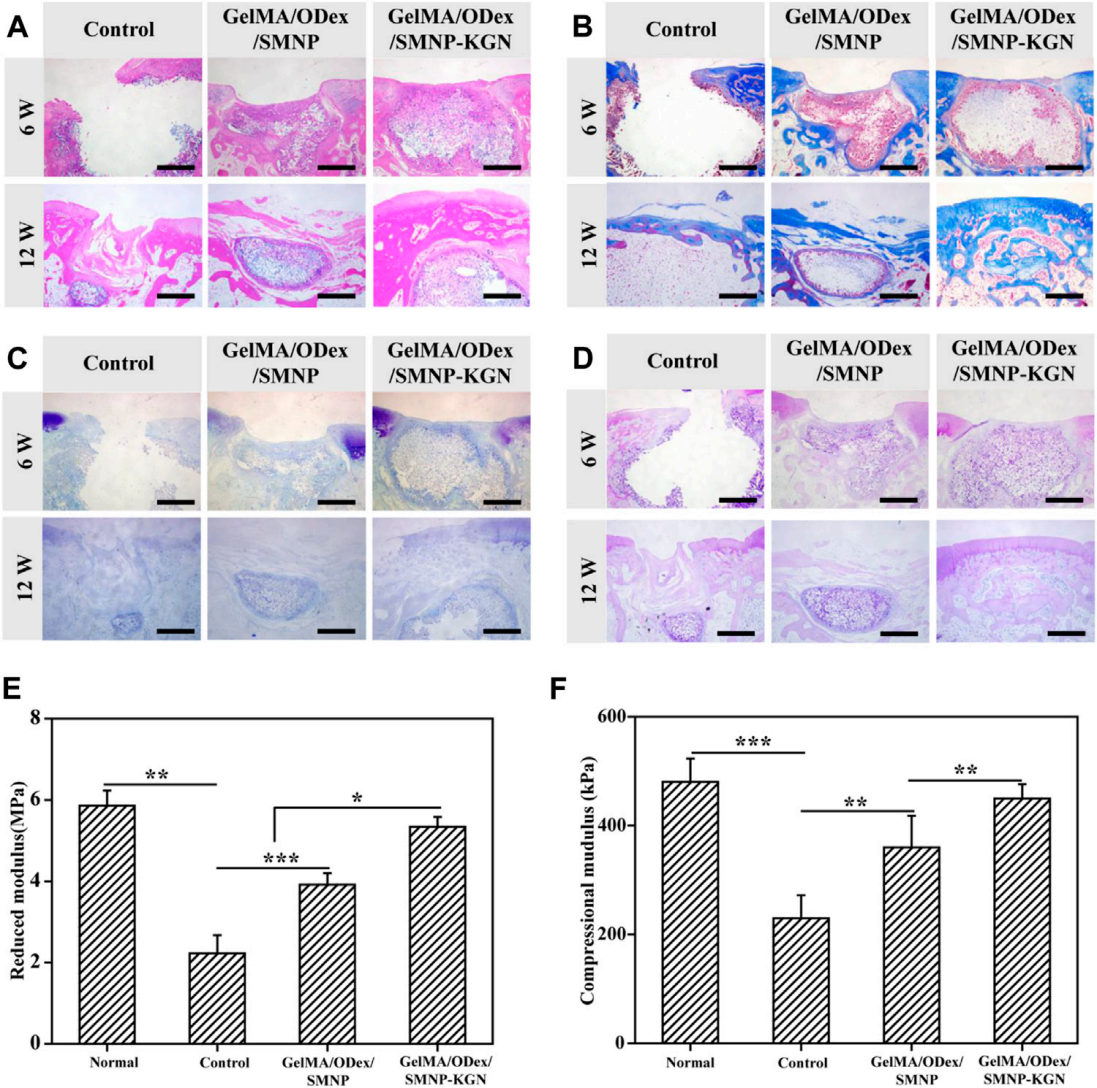
Evaluation of the effect of cartilage regeneration *in vivo*. **(A)** H&E, **(B)** Masson, **(C)** toluidine, and **(D)** PAS staining of defect cartilage at 6 and 12 weeks. Scale bar = 200 μm. **(E)** Reduced modulus and **(F)** compressional modulus of regenerated cartilage in 12 weeks.

The therapeutic effect of the GelMA-ODex/SMNP-KGN hydrogel was further evaluated through histological staining. At the 6th week, an obvious fracture surface was observed in the blank control group by H&E staining (Figure 7A). Despite the occurrence of improvement in the production of cartilage matrix in the GelMA/ODex/SMNP group, the discontinue feature existed all the time. The GelMA-ODex/SMNP-KGN group was mostly filled with cartilage matrix without observed inflammation. At the 12th week, the control group still retained the defect state, verifying the truth that cartilage lacked the innate ability to mount enough healing response. Nevertheless, it was interesting to find that the GelMA-ODex/SMNP group seemed to contain remarkable matrix filling compared to the control group. But the regenerated tissue appeared different and displayed distinct boundaries from the surrounding normal hyaline cartilage. As for the GelMA-ODex/SMNP-KGN group, the regenerated cartilage matrix coincided with normal cartilage matrix and remarkably recovered the cartilage defect flushing with the native tissue.

In 12 weeks of Masson staining, the GelMA/ODex/SMNP-KGN hydrogel treated group showed less collagen deposition than the other two groups (Figure 7B). But PAS and toluidine blue (TB) staining images showed that the experimental group had obvious glycosaminoglycan deposition (Figures 7C,D). At the 12th week, discontinuous sections were hardly observed in the GelMA/ODex/SMNP-KGN hydrogel treated group, which showed abundant new collagen consistent with that of the surrounding normal cartilage. Although the GelMA/ODex/SMNP hydrogel treated group was filled with partial matrix, it seems to be different with normal tissue, which can be ascribed to an inflammation response. The blank group still showed incomplete cross-sections.

To verify the functional recovery of regeneration cartilage, the mechanical properties were evaluated (Figures 7E,F). It was found that the reduced modulus and compressional modulus of the experimental group were higher than those of the other groups and were close to the strength of normal cartilage tissue, which was consistent with the histological results. Meanwhile, it is found that the blank group cannot support the role of force in the empty state, while the GelMA/ODex/SMNP hydrogel treated group will form fibrotic cartilage.

The reason for the better repair effect of the GelMA/ODex/SMNP hydrogel treated group may be the SMNP-KGN nanoparticles carried in the hydrogel. SMNP-KGN nanoparticles was gradually released from hydrogel during its degradation process as shown in MRI results, and KGN was released to the defect tissue under the action of weaker amide bonds. The released KGN could recruit BMSC cells to the defect site, further inducing differentiation into chondrocytes, and making up for the defect of hyaline cartilage, and accelerating cartilage matrix deposition and remodeling.

## Conclusion

In summary, an injectable GelMA/ODex/SMNP-KGN hydrogel was successfully prepared. This kind of hydrogel can be injected to fill damaged cartilage *in situ*, which solves the problem that the scaffold cannot adapt to the shape of the defect. In addition, SMNP-KGN nanoparticles, which have both MRI contrast and cartilage treatment functions, were loaded in hydrogel to realize non-destructive monitoring of the injured site, and recruit surrounding BMSCs to differentiate into chondrocytes to form hyaline cartilage. The histological results further showed that cartilage defect was regenerated significantly at the 12th week after GelMA/ODex/SMNP-KGN hydrogel injection, and the defect edge almost disappeared compared to adjacent normal cartilage tissue. In addition, the mechanical property of regenerated hyaline cartilage was similar to that of normal cartilage. This hydrogel with integrated diagnosis and treatment function will provide a new method for clinical treatment of articular cartilage injury diseases.

## Data Availability

The raw data supporting the conclusions of this article will be made available by the authors, without undue reservation.
